# Rudolf Nieuwenhuys’s later studies in neuroanatomy and functional neuroimaging

**DOI:** 10.1007/s00429-025-02918-4

**Published:** 2025-04-18

**Authors:** Robert Turner

**Affiliations:** Max Planck Institute for Human Cognitive and Brain Mapping, Leipzig, Germany

In 2010 my neuroanatomist colleague Stefan Geyer and I agreed that our MRI studies at the high field strength of 7 tesla now showed promise in delineating some features of the myelination of human cortical grey matter in living human brains (Geyer 2011). On this basis, we decided to produce a book, entitled Microstructural Parcellation of the Human Cerebral Cortex (Geyer 2013), summarizing progress over the last century towards the objective in-vivo parcellation of individual human brains. It was widely recognized that such a parcellation would immediately improve the anatomical identification of the neural substrates involved in any particular functional brain mapping study, and thus enable more realistic systems-level modelling of active neural processes.

Among the prospective authors to whom we reached out was Professor Nieuwenhuys, who was already retired and living in his house in the village of Abcoude, a few kilometers south of the Amsterdam Medical Centre. We invited him to provide a summary of the myeloarchitectural work of Oskar and Cecile Vogt, conducted in Germany between 1903 and 1954, with which he was already very familiar. Once contact had been established, he quickly agreed to help us, and he decided also to take on the larger challenge of reviewing the entire international literature arising from the Vogts’ work on human myeloarchitecture. The result was a superb piece of scholarship, published in our book and in the journal Brain Structure and Function (Nieuwenhuys 2013), which has opened the eyes of many systems neuroscientists to the MRI-observable fine structure in human cortex. We next invited him to give a short course in neuromorphology at our Max Planck Institute for Human Cognitive and Brain Sciences in Leipzig, which he provided with great skill and zest, starting each lecture with a Bach piano piece played by a talented post-doc from the Institute. We recorded these exceptional lectures and transcribed them with their telling illustrations to form the published book: Towards a New Neuromorphology (Nieuwenhuys and Puelles, Springer, 2016).

As a further outcome, Prof. Nieuwenhuys invited me to participate in his series of home seminars, known as the Abcoude Institute of Advanced Studies (AIAS), well described by Desfilis and Medina (2017). The group initially included Pierre-Louis Bazin, Cees Broere, Max Keuken and Birte Forstmann, and met twice yearly from May 2015 until terminated by the onset of the Covid pandemic in 2020. These seminars provided a powerful channel of neuroanatomical understanding, correlating histology with structures observed in living brain while considering the new insights regarding the experience-driven nature of progressive myelination (Fields 2015). Beyond and more, these seminars demonstrated Prof. Nieuwenhuys’s remarkable intellectual generosity, hospitality and personal kindness, and indeed represented a timeless ideal for fruitful scholarly interaction. I often stayed in his house on these occasions, thanks to the kindness of his partner Suzanne Bakker, and came to know of their abiding interest in chamber music, which included annual visits to the Cerne Abbas Music Festival.

Further work inspired and informed by the AIAS seminars include my reviews on the usefulness of high field MRI in neuroscience research (Turner 2017) and the bootstrapping role of myelination in cognitive development (Turner 2019), and also the magisterial neuroanatomical work of Forstmann’s laboratory resulting in their unified 3D map of microscopic architecture and MRI of the human brain (Alkemade 2022).

Seven of Rudolf’s final ten papers published in the last decade were inspired and shaped by AIAS discussions. These culminated in his final work (Nieuwenhuys and Glasser, 2024), a collaboration with Matt Glasser comparing in detail the Vogts’ myeloarchitectonic cortical parcellation with that of Glasser and van Essen based on in-vivo neuroimaging data. This shows how far research has come, but also how much work still needs to be done to relate systems-level brain electrical activity mechanistically to its neural substrates. As MRI techniques and further experimental insights into cortical inhibitory microcircuits continue to progress, Prof. Nieuwenhuys’s late work will likely comprise the firm underpinnings of a greatly enhanced understanding of cortical function.

## Data Availability

No datasets were generated or analysed during the current study.
